# Supplementary material for: RhoA Is a Crucial Regulator of Myoblast Fusion
Source: Cells. 2023 Nov 21;12(23):2673. doi: 10.3390/cells12232673 (PMC10705320; doi:10.3390/cells12232673)
Supplement: Supplementary file 1 [file cells-12-02673-s001.zip › cells-2707113-supplementary.pdf]

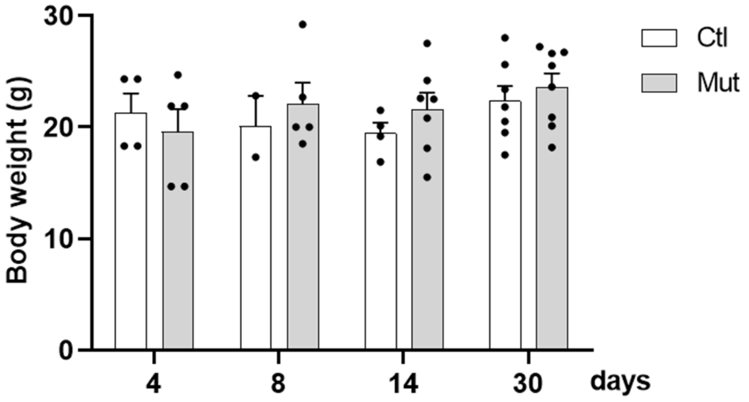

Figure S1. RhoA loss within SCs does not affect mice body weight. Body weight of Tam injected or not mice before, four, eight, fourteen and thirty days after CTX injection (n=2-8).

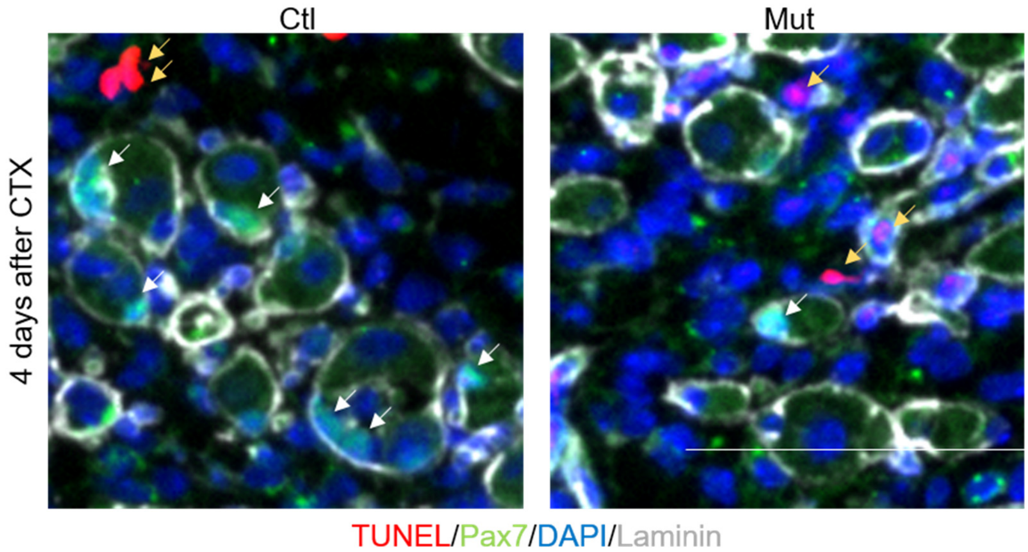

Figure S2. Reduced number of SCs in RhoA-deleted muscle is not due to their aberrant apoptosis. Representative image of *TA* muscle section of Tam or not injected mice immunostained for Pax7 (green), laminin (gray), TUNEL (red) and nuclear staining with DAPI four days after CTX injection. Scale bar 100µm.

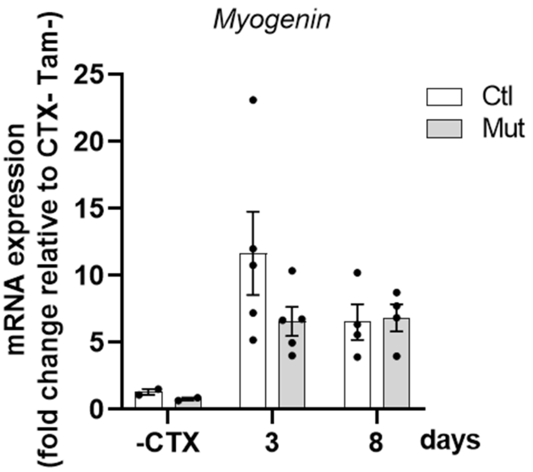

Figure S3. Adult muscle differentiation is not impaired with SCs RhoA deleted Analysis of *Myogenin* mRNA expression by RT-qPCR in Tam injected or not mice before, three and eight days after CTX injection (n=2-5).

A

Symbol	Day0 Expr p-value	Day0 Expr Fold Change	Day1 Expr p-value	Day1 Expr Fold Change	Day3 Expr p-value	Day3 Expr Fold Change
Chrd	3,22E-02	-1,38	4,04E-02	-1,357	4,08E-02	-1,356
Olfr881	2,59E-02	-1,102	3,30E-03	-1,15	4,81E-02	-1,088
C920021L13Rik	1,55E-02	1,256	4,23E-02	1,202	2,53E-02	1,23
Ccdc180	1,08E-02	1,298	3,55E-02	1,227	4,49E-02	1,214
Dhrs2	1,88E-02	1,193	2,54E-02	1,18	2,48E-02	1,181
Fate1	1,03E-03	1,26	5,98E-03	1,196	4,92E-03	1,203
Rsl1 (includes others)	1,76E-02	1,371	2,81E-02	1,332	9,15E-03	1,428
Pear1	1,87E-02	1,363	8,17E-03	1,434	1,49E-02	1,382
Esco1	3,73E-02	2,015	2,61E-02	2,135	2,21E-02	2,194
Gsdmc	1,70E-02	1,289	1,14E-03	-1,239	3,69E-03	-1,199
Ifna4	1,16E-02	1,216	1,60E-02	1,615	1,66E-02	-1,198
Il12rb1	1,23E-02	1,151	2,09E-02	-1,135	3,28E-02	1,122
Lilrb3	1,07E-02	1,201	4,27E-02	-1,148	6,24E-03	-1,223
Vmn1r192 (includes others)	3,38E-03	-1,179	5,02E-03	1,229	1,83E-03	-1,137
Vmn2r32 (includes others)	1,32E-02	1,555	2,62E-02	1,289	1,42E-02	-1,33
Adam20	2,16E-02	-1,206	2,87E-02	-1,106	1,74E-02	1,118
AI987944 (includes others)	8,46E-04	-1,447	3,57E-02	-1,955	2,63E-02	1,666

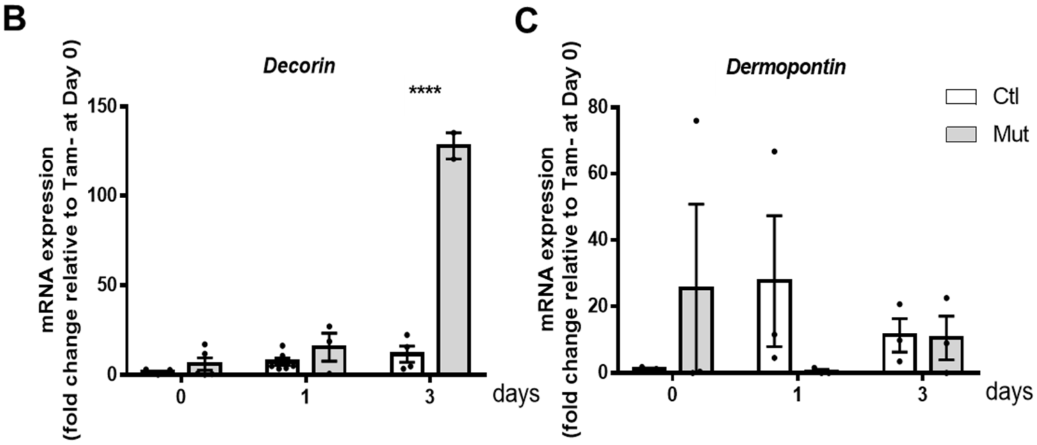

Figure S4. The absence of RhoA in SCs affects some expression genes. **(A)** Top 17 genes identified by IPA whose expression is RhoA dependent and is deregulated in all the differentiation state. **(B)** Analysis of *Decorin* (*Dcn*) and **(C)** *Dermopontin (Dpn)* mRNA expression by RT-qPCR in FACS-sorted SCs control or RhoA-deleted, cultured in rich medium (Day 0) or 1 (Day 1) and 3 (Day 3) after differentiation. Data were normalized by *Hmbs* expression and relative to Tam- at Day 0 (n=2-6). Means ± SEM **** $P < 0.0001$ (ordinary two-way ANOVA with Sidak's test).
